# Nose-Flap Devices Used for Two-Stage Weaning Produce Wounds in the Nostrils of Beef Calves: Case Report

**DOI:** 10.3390/ani12111452

**Published:** 2022-06-04

**Authors:** Tiago S. Valente, Lucas R. B. Ruiz, Fernanda Macitelli, Mateus J. R. Paranhos da Costa

**Affiliations:** 1 Department of Agricultural, Food and Nutritional Science, University of Alberta, Edmonton, AB T6G-2H1, Canada; 2 Departamento de Zootecnia, Faculdade de Ciências Agrárias e Veterinárias, Universidade Estadual Paulista, Jaboticabal 14884-900, SP, Brazil; 3 Programa de Pós-Graduação em Zootecnia, Faculdade de Ciências Agrárias e Veterinárias, Universidade Estadual Paulista, Jaboticabal 14884-900, SP, Brazil; lksruiz.lr@gmail.com; 4 Instituto de Ciências da Saúde, Universidade Federal do Mato Grosso, Sinop 78550-000, MT, Brazil; fernandambenez@gmail.com; 5 Conselho Nacional de Desenvolvimento Científico e Tecnológico, Brasília 71605-001, DF, Brazil

**Keywords:** animal welfare, anti-suckling device, cow-calf operation, nasal abrasions

## Abstract

**Simple Summary:**

It is often mentioned that calf stress is reduced when a two-stage weaning method is adopted. However, is this always the case? Here, we describe the occurrence of negative effects on the nostrils of calves from the use of nose-flap devices to aid weaning, which can lead to health and welfare impoverishment. Commercial nose-flap devices were fitted in the nostrils of 21 pure and 20 crossbred Nellore calves for five days. Nose-flap retention was 73.2%. Without exception, all calves showed open wounds in the nasal septum, including those that lost the device before day five. Almost half of the calves showed weight loss during the period wearing the nose-flap devices. Due to the incidence of nasal-septum injuries, it is imperative that more studies be done to assess these overlooked effects of the two-stage weaning method, which is referred to as a low-stress practice for beef calves.

**Abstract:**

This case report aims to describe the occurrence of negative impacts of wearing nose-flap devices on beef calves subjected to the two-stage weaning method. Forty-one calves, twenty-one pure Nellore and twenty F1 Angus-Nellore, were weaned on average at 236 days of age. Commercial nose-flap devices were fitted in the nostrils of the calves (d0) to prevent suckling and removed five days later (d5). Individual body weights were assessed at d0 and d5, and average daily gain (ADG) was calculated. At d5, during nose-flap device removal, it was noted that 26.8% of the calves lost the nose-flap device; however, all of them had wounds in their nostrils (no injuries in the nostrils had been observed on d0). To assess the severity of these injuries, an impairment score was assigned to each calf, ranging from 1 = no lesions to 5 = injured with purulent discharge. A logistic regression model was fitted to evaluate the effect of sex and genetic group on nose-flap retention (kept or lost). The retention rate did not differ (*p* > 0.05) between sex and genetic groups. All calves showed at least open wounds of the nasal septum (score 2), including those that lost the nose-flaps before d5. Almost half of the calves showed weight loss during this period. We conclude that there is a considerable risk of the two-stage weaning method compromising the physical integrity of the nostrils of beef calves through the use of these devices, and due to this, it should not be referred to as a low-stress weaning practice for beef calves.

## 1. Introduction

It has been well documented that the abrupt weaning (when cow-calf pairs are suddenly separated from each other) is a stressful process for beef cattle, leading to negative impacts on the health, welfare, and performance of both cows and calves [1,2,3]. Therefore, a number of studies have been carried out looking for alternative weaning strategies, such as the two-stage weaning method [4,5,6].

The first stage of the two-stage weaning method is characterized by keeping the calves with their mothers while wearing a nose-flap device to prevent suckling. The duration of this stage varies considerably among studies, lasting from three [4] to twenty-one days [7,8]. The second stage is carried out when cows and calves are permanently separated after removing the nose-flap device. It is assumed that this weaning method reduces the negative synergistic effect caused by the interruption of milk feeding and the loss of social interactions and physical contact with their mothers. According to previous studies, calves wearing a nose-flap device spend more time eating/grazing and less time walking and vocalizing, besides showing a higher post-weaning average daily gain (ADG) than those abruptly weaned [4]. These results have been fully or partially confirmed by other studies [9,10,11], reinforcing the concept that the two-stage method offers an alternative for the low-stress weaning of beef calves.

Although two-stage weaning is considered a low-stress method by some farmers and researchers, there is still no consensus in the scientific literature about the overall stress associated with this method, the short and long-term negative effects on immune responses and health, as well as the emotional frustration caused to the calves wearing the nose-flap device and the economic implications of this method [1,2,5,8,9,12]. According to Enriquez et al. [5], this weaning practice does not reduce overall stress but redistributes it over two stages. Additionally, little attention has been paid to the evidence that there is a high risk of nasal wounds, resulting in nasal-septum open lesions, as reported by Taylor et al. [3] and Lambertz et al. [13]. Besides the primary immune response being negatively affected, calves wearing nose devices are inevitably exposed to pain and infectious agents, which could lead to health problems, such as traumatic rhinitis and a pituitary abscess that, in some cases, results in death [8,14,15,16,17].

According to Tucker et al. [12], determining the best non-abrupt weaning practices and identifying the effects on calf health were considered a priority for future beef cattle welfare-related research in the USA. However, little attention has been paid to the occurrence of nasal-septum impairment. To the best of our knowledge, there are few studies assessing the negative effects of the two-stage weaning method on the calves’ health and overall welfare. Thus, the aim of this case report is to provide information about the potential negative impact of wearing a commercial nose-flap device on the nostrils of beef calves as well as ADG when subjected to the two-stage weaning method.

## 2. Materials and Methods

Observations were carried out at Agropecuária Orvalho das Flores, a private farm located at the municipality of Araguaiana, Mato Grosso State, Brazil. The work was approved by the Committee on Ethics in the Use of Animals of Araguaia, Federal University of Mato Grosso, UFMT, Campus Araguaia (certified n. 23108.037555/2020-66).

**Figure 1:**
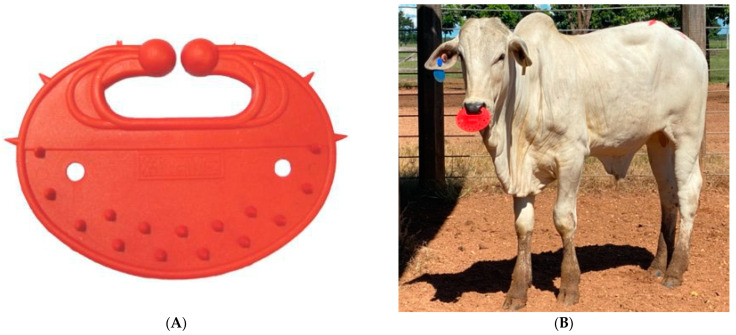
Photograph of the plastic nose-flap device (WalMur^®^, Brazil) used in this study (**A**); and a purebred Nellore calf wearing a plastic nose-flap device (**B**).

The two-stage weaning method was applied to 41 pure and crossbred Nellore calves, 21 purebred Nellore (NE = 11 males and 10 females), and 20 F1 Angus-Nellore (AN = 11 males and 9 females) at 236 ± 4 days of age on average. At d0, all calves were restrained in the squeeze chute, weighed, and weaned by fitting a commercial plastic nose-flap device (WalMur^®^, Brazil) in their nostrils (Figure 1). The devices used were 13.5 cm × 10 cm. After finishing these handling procedures, cows and calves were driven to the pasture and kept together for five days, with free access and high availability to good quality (good nutrition value) grass, water, and mineral supplement. At d5, all calves were permanently separated from their dams after being processed in the squeeze chute, weighed, and the nose-flap removed.

The nasal septum of calves was assessed at d0 and d5, ascribing one of the following grades of nasal-septum impairment: (1) no lesions, (2) nasal septum injured without bleeding or secretion, (3) nasal septum injured with bleeding, (4) nasal septum injured with translucent secretion, or (5) nasal septum injured with purulent secretion. This method was developed based on the Welfare Quality protocol for beef cattle [18], taking into consideration the description of Carbonell [19] for nasal discharge, and was performed by only one observer. Nose-flap retention ratio was calculated, and body weights assessed at d0 and d5 were used to calculate individual ADG during the period of wearing nose-flap devices.

### Statistical Analysis

A logistic regression model was fitted to evaluate the effect of sex and genetic group on nose-flap retention, considering a binomial distribution for the response variable (kept x lost the device before d5). Statistical analyses were carried out using the R software (v. 4.0.5; R Core Team, Wien, Austria, 2018). Results were considered significant when the *p*-value was ≤ 0.05.

## 3. Results and Discussion

### 3.1. Nose-Flaps Retention

The nasal flap retention rate at d5 in this study was 73.2% (*n* = 30/41). This value is slightly lower than the retention rate of 76.4% reported by Lambertz et al. [13] for 89 German Angus and Simmental purebreds and crossbred calves. Higher retention rates (95% or greater) were also reported by Haley et al. [4] for crossbred calves (primarily Charolais, Hereford, and Simmental breeds) when exploring possible advantages of the two-stage weaning over four trials. Similarly, Taylor et al. [2] reported that only one calf lost the device before removal. It is likely that these differences in the retention rate between studies are dependent on the breed, age of weaning, the number of days wearing the device, and the design of the nose-flap used [6,13].

In the present study, it was not possible to determine under what circumstances or when the calves lost their nose-flaps, except for one single NE heifer-calf, which removed the nose-flap twice immediately after fixing it (not considered in the data analyses). However, it should be noted that approximately one-third of the animals lost their devices, which indicates a considerable inefficiency of this weaning method when applied to purebred Nellore and F1 Angus-Nellore calves. It is worth mentioning that the retention rate was numerically different between sex and genetic groups but not significant (*p* > 0.05). In this case, the highest percentage of nasal flap loss was observed for AN heifer calves (3 of 9, 33%), followed by NE heifer calves (3 of 10, 30%), NE bull calves (3 of 11, 27%) and AN bull calves (2 of 11, 18%).

Since the device is fitted to prevent calves from suckling before being completely separated from their mothers, the retention rate is an important indicator of the weaning method efficiency. Calves that lost their nose-flaps were probably not deprived of milk consumption during the first stage of weaning and, therefore, they were probably more stressed when physically separated from their mothers, similar to what occurs when abrupt weaning is carried out [1,2].

### 3.2. Open Wounds in the Nasal Septum and Potential Complications

**Figure 2:**
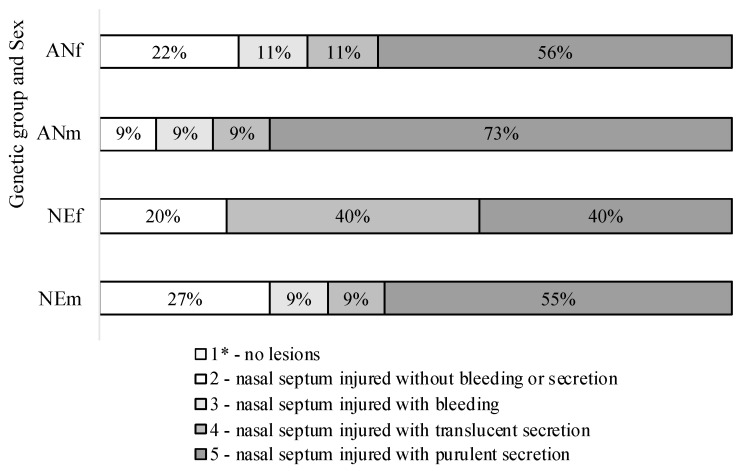
Percentage of calves classified in each grade of nasal septum impairment at device removal day (d5) according to genetic group and sex, in which: ANf = F1 Angus-Nellore female (*n* = 9); ANm = F1 Angus-Nellore male (*n* = 11); NEf = Nellore female (*n* = 10); NEm = Nellore male (*n* = 11). * No calves received score 1 after the removal of nose-flap devices.

**Figure 3:**
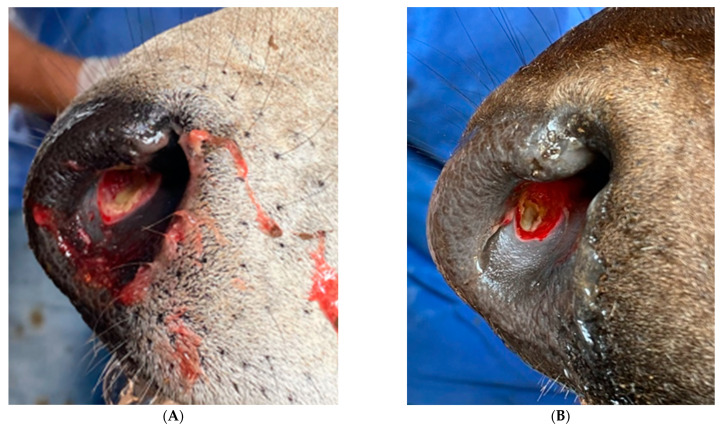
Nasal-septum injuries in a purebred Nellore ((**A**), score 5) and an F1 Angus-Nellore ((**B**), score 4) calf.

No injuries in the calves’ nostrils were observed on d0, and a score of nasal-septum impairment equal to 1 was assigned to all 41 animals. However, by d5, all calves showed open wounds in their nasal septum, including those that lost the nose-flap devices before being handled at d5 (Figure 2). The wound was characterized as an abrasion caused by the skin rubbing against the nose-flap device, as shown in Figure 3. The wounds could be aggravated due to the calf’s behavior if trying to remove the device [19]. An extreme situation was observed in one of the AN bull calves, which had a perforated septum and the highest weight loss (−4.0 kg/day).

The occurrence of extensive ulcerative lesions in the nasal septum resulting from wearing nose-flap devices was already reported by Taylor et al. [3], Lambertz et al. [13], and Fernandes et al. [14]. Surprisingly, although the authors clearly reported the occurrence of physical injuries, with Fernandes et al. [14] indicating that the health of more than 1% of the calves was heavily impacted by pituitary abscesses, the short- and long-term negative side effects of this weaning method are not properly addressed in the literature. Thus, as suggested by Tucker et al. [12], there is a need for more research focusing on weaning methods aiming to reduce overall stress while improving the welfare of beef calves. Additionally, to the best of our knowledge, all studies carried out on two-stage weaning were conducted with British and Continental cattle breeds, lacking studies to evaluate or validate this weaning method in *Bos indicus* cattle [6].

Lambertz et al. [13], who first developed a scoring system to evaluate injuries caused by nose-flap devices, reported that only 3.8% of the calves lacked visible irritations in their nasal septum immediately after device removal. The authors also reported that 30% of the injured calves showed heavy bleeding, and 10% suffered from severe wounds on the day of device removal. In addition, almost 45% of calves were suffering from injuries one week after anti-suckling device removal, showing signs of local inflammation. Taylor et al. [3] reported similar results, indicating the occurrence of hemorrhage, ulcers, and nasal erosions at the time of device removal, classifying them as modest-to-moderate injuries. Despite reporting the occurrence of injuries, which impoverish calf health and welfare, no effort was made by the authors to record the severity and for how long the animals were suffering from nasal injuries. Additionally, these authors [3,13] focused their conclusion on animal performance, indicating the use of nose-flaps negatively impacted calf weight gain, corroborating our results and those from previous studies [4,8,20].

It can be assumed that exposing the nasal septum skin to such harmful stimulus will surely lead to an unpleasant sensation of pain [21]. However, despite the high occurrence of nasal wounds reported in different studies and the potential risk of calf health and welfare impoverishment, none of the authors recommended caution on the use of nose-flap devices and classification of their use in two-stage weaning as a low-stress method. Perhaps surprisingly, some authors did suggest that the nose-flap material, design, brand, and the number of days wearing the device need to be modified to minimize nasal injuries [3,6,13]. This is possibly questionable since distinct materials, design, and the number of days wearing anti-suckling devices resulted in the occurrence of nasal injuries across different studies for male and female calves of British and Continental breeds.

Although not assessed, it is possible to assume that the wounds caused by wearing nose-flap devices resulted in pain, which can be characterized as an additional stressor to this weaning method. Alongside the occurrence of local wounds, the use of nose-flaps may be a gateway for infections, leading the calves to face other health problems. The wounds can progress to traumatic bacterial rhinitis, which, in severe cases, could lead to pituitary abscess syndrome [14,15,16]. According to Loretti et al. [15], the first clinical signs of pituitary abscess syndrome were observed approximately 12 to 60 days after fitting the nose-flap devices in affected calves. Additionally, Lippolis et al. [8] reported that the two-stage weaning method, using the nose-flaps for 21 consecutive days, resulted in decreased antibody titer response following vaccination and ADG, indicating that the nose-flap devices may induce an underlying physiological stress response, with negative effects on the general performance after weaning of Angus, Hereford, and Angus × Hereford calves.

### 3.3. Effects of Nose-Flap Devices on Calf Weight Gain

In our study, the average initial body weight was 209 ± 24 and 259 ± 28 kg for NE and AN calves, respectively. Almost half of the calves reduced body weight (46%, 19/41) during the five days of wearing nose-flaps, and the overall mean ADG was −0.17 ± 1.29 kg/d (ranging from −4.0 to 2.6 kg/d). These results corroborate the results of Taylor et al. [3], who also observed a negative impact of the two-stage weaning method on calf ADG for 7 and 28 days after weaning. However, Burke et al. [20] reported that this negative impact has only a short-term effect on ADG, which is reduced by compensatory post-weaning weight gain after nose-flap removal.

Calves that lost the nose-flap device before d5 were also the animals with higher weight loss (−0.33 ± 1.35 kg/day) compared to those that remained with the device for five consecutive days (0.29 ± 1.01 kg/day). The lower performance level can be explained by the fact that all of them presented open wounds in their nasal septum, and some of them (3/11) reached the higher grade of nasal-septum impairment, showing purulent discharge. Although it is not possible to determine for how long each calf was wearing the anti-suckling device, it is reasonable to assume that even when they were worn for a short time, the devices were probably a cause of physical and mental stress, negatively affecting calf welfare and leading to impoverished performance.

### 3.4. General Considerations

Despite some authors considering that the two-stage weaning method reduces the stress responses of cows and calves by mimicking the natural weaning process [1,4,10,22], most of them did not measure or mention the occurrence of nasal lesions and conclude that this management practice is better when compared to the abrupt weaning method. This assumption was mainly based on the fact that calves weaned by the two-stage method exhibited less vocalization and walking activity [4,23] and spent more time eating and resting [4].

Other authors highlighted that despite the positive effects on calf behaviors, the use of nose-flaps leads to physical and emotional negative consequences characterized by lesions in the nasal septum [3,13] and frustration [12]. Moreover, calves weaned by the two-stage method are exposed to additional stressors, including fitting and removing the nose-flaps, followed by the permanent separation from their mothers, splitting overall stress into two or more bouts over a short period of time [5].

Although behavior assessment and biomarker analysis were not employed in the present study, it is well documented that the calves must deal with many nutritional, physiological, and emotional challenges during the weaning process. Thus, all these challenges must be carefully controlled, ensuring as low a stressful situation as possible for the calves. Recently, Taylor et al. [3] reported that it is improbable that any alteration in the two-stage weaning protocol would confirm it as a low-stress method, mainly because calves submitted to this procedure had poorer weight gain both pre-and post-weaning.

The high occurrence of severe injuries identified in this case report corroborates the findings of Taylor et al. [3]. Thus, according to our results and those that reported occurrence of injuries [3,13,14], there is a considerable risk of nose-flap devices leading to negative effects on calf health and welfare. Due to this, the short-term benefits of two-stage weaning are questionable. Moreover, to the best of our knowledge, there is no information available in the scientific literature about the consequences of wearing nose-flap devices in purebred Bos indicus cattle and crosses [6]. Considering the magnitude of the Brazilian beef industry and the number of calves weaned annually, it is imperative to evaluate and validate different weaning strategies for Bos indicus cattle breeds.

## 4. Conclusions

The present case report revealed a high occurrence of nasal septum injuries caused by the nose-flap devices when implementing two-stage weaning, which probably leads to pain and suffering. Our findings corroborate what was reported by other researchers using different device materials and designs, as well as a different number of days wearing nose-flaps. There is a risk of this two-stage weaning method compromising calf health and impoverishing their overall welfare. Thus, we conclude that it is important to adopt the precautionary principle, assuming the two-stage weaning method should not be considered a low-stress management practice for weaning beef calves. Thus, additional studies are crucial to carefully evaluate the short and long-term effects of this weaning method on calves’ health and welfare, taking into account behavior traits, the occurrence of injuries, physiological parameters, inflammatory biomarkers, and blood metabolites.

## Data Availability

Not applicable.

## References

[1] Enríquez D.H., Hötzel M.J., Ungerfeld R. (2011). Minimising the stress of weaning of beef calves: A review. Acta Vet. Scand..

[2] Lynch E., McGee M., Earley B. (2019). Weaning management of beef calves with implications for animal health and welfare. J. Appl. Anim. Res..

[3] Taylor J.D., Gilliam J.N., Mourer G., Stansberry C. (2020). Comparison of effects of four weaning methods on health and performance of beef calves. Animal.

[4] Haley D.B., Bailey D.W., Stookey J.M. (2005). The effects of weaning beef calves in two stages on their behavior and growth rate. J. Anim. Sci..

[5] Enríquez D.H., Ungerfeld R., Quintas G., Guidoni A.L., Hötzel M.J. (2010). The effects of alternative weaning methods on behaviour in beef calves. Livest. Sci..

[6] Orihuela A., Mota-Rojas D., Fabio Napolitano F. (2020). Weaning strategies to improve productivity and animal welfare in zebu (*Bos indicus*) and water buffaloes (*Bubalus bubalis*). J. Anim. Behav. Biometeorol..

[7] Alvez P., Quintans G., Hötzel M.J., Ungerfeld R. (2016). Two-step weaning in beef calves: Permanence of nose flaps for 7 or 21 days does not influence the behaviour response. Anim. Prod. Sci..

[8] Lippolis K.D., Ahola J.K., Mayo C.E., Fischer M.C., Callan R.J. (2016). Effects of two-stage weaning with nose flap devices applied to calves on cow body condition, calf performance, and calf humoral immune response. J. Anim. Sci..

[9] Hötzel M.J., Quintans G., Ungerfeld R. (2012). Behaviour response to two-step weaning is diminished in beef calves previously submitted to temporary weaning with nose flaps. Livest. Sci..

[10] Ungerfeld R., Quintans G., Hötzel M.J. (2016). Minimizing cows’ stress when calves were early weaned using the two-step method with nose flaps. Animal.

[11] Rauch J.C., Stokes R.S., Shike D.W. (2019). Evaluation of two-stage weaning and trace mineral injection on receiving cattle growth performance and behavior. Transl. Anim. Sci..

[12] Tucker C.B., Coetzee J.F., Stookey J.M., Thomson D.U., Grandin T., Schwartzkopf-Genswein K.S. (2015). Beef cattle welfare in the USA: Identification of priorities for future research. Anim. Health Res. Rev..

[13] Lambertz C., Bowen P.R., Erhardt G., Gauly M. (2015). Effects of weaning beef cattle in two stages or by abrupt separation on nasal abrasions, behaviour, and weight gain. Anim. Prod. Sci..

[14] Fernandes C.G., Schild A.L., Riet-Correa F., Baialardi C.E.G., Stigger A.L. (2000). Pituitary abscess in young calves associated with the use of a controlled suckling device. J. Vet. Diagn. Investig..

[15] Loretti A.P., Ilha M.R., Riet-Correa G., Driemeier D., Colodel E.M., Barros C.S.L. (2003). Pituitary abscess syndrome in calves following injury of the nasal septum by a plastic device used to prevent suckling. Pesqui. Vet. Bras..

[16] Câmara A.C.L., Borges J.R.J., Godoy R.F.D., Moscardini A.R., Mustafa V.D.S., Castro M.B.D., Drummond V. (2009). Pituitary abscess syndrome in calves from Mid-Western Brazil. Pesqui. Vet. Bras..

[17] Galiza G.J., Silva M.L., Dantas A.F., Simões S.V., Riet-Correa F. (2010). Diseases of the nervous system of cattle in the semiarid of Northeastern Brazil. Pesqui. Vet. Bras..

[18] Welfare Quality^®^ Welfare Quality^®^ Assessment Protocol for Cattle. Welfare Quality^®^ Consortium, Lelystad, The Netherlands. https://www.google.com.tw/url?sa=t&rct=j&q=&esrc=s&source=web&cd=&ved=2ahUKEwigh5fK2JL4AhWgQjABHQnuD-gQFnoECAgQAQ&url=https%3A%2F%2Fedepot.wur.nl%2F233467&usg=AOvVaw037V3tHrF005vMqX7GyAXe..

[19] Carbonell P.L. (1979). Bovine nasal granuloma: Gross and microscopic lesions. Vet. Pathol..

[20] Burke N.C., Scaglia G., Boland H.T., Swecker W.S. (2009). Influence of two-stage weaning with subsequent transport on body weight, plasma lipid peroxidation, plasma selenium, and on leukocyte glutathione peroxidase and glutathione reductase activity in beef calves. Vet. Immunol. Immunopathol..

[21] Millan M.J. (1999). The induction of pain: An integrative review. Prog. Neurobiol..

[22] Weary D.M., Jasper J., Hötzel M.J. (2008). Understanding weaning distress. Appl. Anim. Behav. Sci..

[23] Loberg J.M., Hernandez C.E., Thierfelder T., Jensen M.B., Berg C., Lidfors L. (2008). Weaning and separation in two steps—A way to decrease stress in dairy calves suckled by foster cows. Appl. Anim. Behav. Sci..

